# Wellness Tour for Tribal Communities During the COVID-19 Pandemic: Uniting Sacred Space with Western Medicine to Prevent Substance Use

**DOI:** 10.1007/s10900-023-01295-5

**Published:** 2023-10-17

**Authors:** Nausheen Wakhlu, Claradina Soto, Mike Duncan, Albert Titman, Barbara J. Turner

**Affiliations:** 1 Department of Medicine, Keck School of Medicine of University of Southern California, 2020 Zonal Avenue, Los Angeles, CA 90033 United States of America; 2 Department of Population and Public Health Sciences, Keck School of Medicine, University of Southern California, 2001 North Soto Street, Los Angeles, 90032 United States of America; 3 California Consortium for Urban Indian Health, 1016 Lincoln Blvd #111, San Francisco, CA 94129 United States of America; 4 Gehr Center for Health Systems Science, Keck School of Medicine of University of Southern California, 2020 Zonal Avenue, Los Angeles, CA 90033 United States of America

**Keywords:** American Indian, community-based, Substance use disorder, substance abuse education, COVID-19, ceremonial behavior

## Abstract

Substance use disorders (SUD) and overdose deaths worsened further during the Covid-19 pandemic in American Indian and Alaska Native (AIAN) communities. The Native Dad’s Network (NDN) delivered the Wellness Tour, offering cultural activities and SUD prevention education, from March 2021 to June 2022, to 11 AIAN tribal communities across California. The in-person program created a “sacred space” through culturally congruent song, dance, and prayer. SUD education included: a lecture about opioids and SUD; group talking circles; an educational skit led by adolescents; and training in naloxone and fentanyl testing strip use along with supplies. After the day-long program, 341 participants agreed strongly on a 5-point Likert type question that it improved their quality of life (mean = 4.7). Among 243 respondents, agreement was strong (mean = 4.8) to two Likert-type questions about cultural relevance and confidence in using naloxone. This AIAN-led program adopted safe practices during the pandemic to deliver culturally congruent SUD prevention education to severely affected AIAN communities.

## Introduction

The US American Indian and Alaska Native (AIAN) population has been disproportionately affected by illicit drug use. The proportions of AIAN persons reporting drug abuse in the past month (17.4%) or year (28.5%) were higher than those of any other racial/ethnic group according to the 2018 National Survey on Drug Use and Health [1]. Recent national data revealed that the AIAN population had the highest mortality rate from drug overdose which was 31% higher than the next highest rate that was for non-Hispanic whites [2]. Non-fatal overdose from opioids and other drugs also greatly threatens the health of AIAN communities [3–5]. Efforts to prevent the dire consequences of substance use disorders (SUD) are critical to reducing morbidity and mortality of the AIAN population.

AIAN persons with or at risk of SUD have structural, social, cultural, and geographical barriers to SUD prevention and treatment [6]. Stigmatizing racial stereotypes and historical trauma have compromised SUD care as well as general health care in a study of indigenous Canadians [7]. Unlike Western medicine, traditional AIAN healing practices focus on integrating cultural social norms, family dynamics, and problem-solving skills [8]. A systematic review of opioid use disorder treatment in rural AIAN communities reported that participants’ priorities included culturally-grounded health and wellness interventions and utilizing community reinforcement approaches [9]. In recent years, SUD prevention programs for AIAN communities have attempted to address these priorities by incorporating culturally appropriate educational methods [9]. Unfortunately, access to in-person prevention programs was restricted during the pandemic. However, studies of virtual SUD programs using computer technology have reported mixed results [10].

The Wellness Tour, a culturally specific community-based program, was launched during the COVID-19 pandemic to address SUD prevention in AIAN communities located in or near California reservations. This program was developed by the Native Dad’s Network (NDN), a non-profit community-based organization in California with five AIAN board members who have been personally affected by substance use in their families. Two board members -- the executive and deputy director–are medication-assisted treatment counselors. The NDN aimed to interrupt the cycle of addiction through in-person programs for AIAN communities that offered Western-style SUD prevention education but in the context of traditional ceremonial practices and culturally congruent, group-based learning methods. The program opened and closed by creating a “sacred space” where the community can gather in a specific area (e.g., outside in nature, a clinic, a community organization) to engage in traditional ways to feel safe, heal, and honor its spiritual well-being. The Wellness Tour also focused on the importance of the indigenous family structure by offering intergenerational healing programs to provide a positive way to restore and reconnect to cultural ways.

This paper describes the development and implementation of the Wellness Tour’s integration of traditional, culturally congruent ceremonial traditions with Western medicine SUD prevention training and resources in AIAN community settings. Results of an anonymous questionnaire completed by program participants offered insights into issues related to illicit drug use and evaluation of the Wellness Tour program. Despite the challenges of conducting an in-person program during the COVID-19 pandemic, the Wellness Tour serves as a valuable model to deliver SUD prevention in diverse tribal communities.

## Methods

### Wellness Tour Overview

From March 2021 through June 2022, NDN leaders traveled to 11 tribal communities across California to engage AIAN youth, families, and community members in SUD recovery with the Wellness Tour. The Wellness Tour visits combined supportive and healing group cultural practices with Western SUD prevention (Table 1). At each event, the NDN created a sacred space by engaging attendees of all ages with traditional singing, dancing, and prayer to reinforce connections among community members. Western medicine-based SUD prevention education was combined with training in the use of medication and testing to reduce the risk of death from opioid overdose [11, 12]. Based on federal recommendations, COVID-19 risk reduction recommendations were followed by holding in-person meetings outside, placing chairs 6 feet or more apart, and wearing masks for closer interactions [13, 14]. The University of Southern California Institutional Review Board approved this project (UP-21-00962).

**Table 1:** Events and core components of the NDN Wellness Tour

Program Event	Activity	Presenters	Traditional or Western medicine activities
Creation of a sacred space (1–2 h)	Native ceremony with song, dance and prayer	Tribal & NDN Leaders	Traditional
Education opioid use, abuse, and risks for AIAN communities (1 h)	PowerPoint presentation with breakout discussion groups	NDN leaders	Blended
Naloxone and fentanyl testing training (1-hour)	Intranasal naloxone and fentanyl testing, fentanyl test strips distributed	NDN leaders	Western
Skit about acting out an overdose (1 h)	Demonstrating how to identify an overdose and emotional impact on community members	Skit by 18–25 y/o community members	Blended
Wellness Circles and testimonials (1–2 h)	Safe space to discuss substance use and how cannabis and other SUD affect families/ communities	Lead by NDN leaders	Blended
Conclusion with sacred space activities (1 h)	NDN leaders perform Head-Shake Dance, and group performs tribal dances, song and prayer	Tribal & NDN Leaders	Traditional

### Engaging Tribes for Wellness Tours

**Fig. 1:**
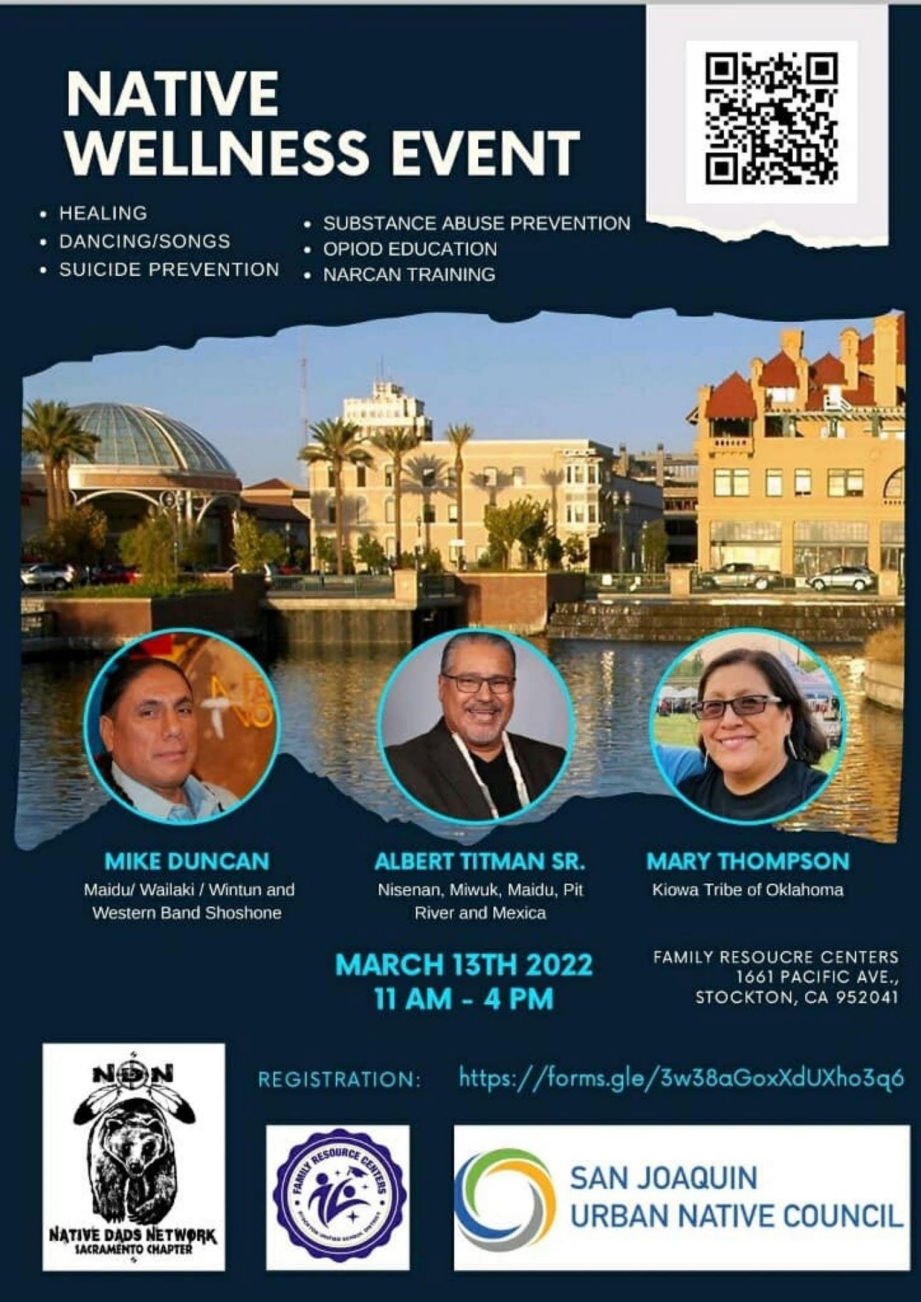
Wellness Tour Facebook Poster

The NDN executive director (MD) reached out to California local tribes to deliver the Wellness Tour to adolescents aged 13 or older and adults. Following cultural traditions, the NDN director met with tribal leaders to learn about their priorities for the program and to obtain permission for the Wellness Tour. Once confirmed and scheduled, promotional materials and information about the Wellness Tour community program were shared through phone calls, email announcements, and Facebook posts (Fig. 1). The announcement described the one-day in-person gathering as focusing especially on awareness, education, and cultural responses to the opioid crisis affecting tribal communities. Topics included opioid education, an overdose skit with naloxone training, testimonials, workshops about SUD prevention, and cultural activities. Incentives for participation included free meals, raffles, children’s activities, and childcare. After the first two NDN Wellness Tour community events, word-of-mouth prompted other tribal representatives to contact the NDN executive director to receive the program in their local tribal area. The Seventh Generation Fund financially supported the first Wellness Tour and subsequent tours were sponsored by the Sierra Health Foundation from funding by the Substance Abuse and Mental Health Services Administration. To supplement a limited budget, several tribes contributed free food and lodging for NDN leaders and their families.

### Creating a Sacred Space

To create a culturally appropriate sacred space with Wellness Tour participants, NDN leaders started and closed each program with spiritually, physically, and emotionally engaged activities including prayers, songs, and dances known to tribal community members. To recognize local customs and traditions. the leaders sought each tribal community’s guidance regarding members to lead these activities. A hand drum, a clapper stick made from a branch of an elderberry tree (idiophone instrument), and/or rattles/shakers made from dried gourds were often used to accompany native song and dance. The NDN leaders were experienced in performing traditional social dances, such as the Head-Shake, where a dancer moves his head to activate a flickered feather forehead band, but dances familiar to the specific tribe were also performed. NDN leaders and the tribal community collaborated in establishing a sense of community and shared culture through the sacred space.

### Opioid Education and Training with Naloxone and Fentanyl Testing

After welcome and traditional activities, NDN leaders presented a 30-minute PowerPoint educational program developed by the California Consortium for Urban Indian Health about opioids, risks, and opportunities to reduce risks. A breakout session followed that included talking circles, typical of tribal meetings, where participants shared testimonials about opioids, other illicit drugs, overdose, and destructive effects on their tribal community.

NDN leaders then offered a demonstration of administering intranasal naloxone as a life-saving intervention for a person who may be experiencing an opioid overdose. They distributed free samples to participants because, despite increasing expenditures for this drug by Indian Health Service since 2016, supplies went primarily to pharmacists and first responders [15]. Training in the use of fentanyl strips was also offered to detect this potentially deadly substance [16]. Free fentanyl test strips were also provided to participants in light of limited supplies for tribal communities.

NDN leaders organized a 30-minute skit in which young AIAN participants (18 to 25 years) role-played an overdose scenario to practice actions to be taken. The multiple roles in the skit included playing the victim, the person who discovered the victim, and community members involved in deciding appropriate actions. Learning points from this emotionally charged skit included how to: assess a victim’s consciousness and respiratory status, stay calm under duress, administer naloxone, and contact emergency services.

### Breakout Discussion Sessions: Wellness Circle

The NDN leaders held a one-hour, open-ended Wellness Circle discussion about SUD topics selected by the community such as alcohol use and abuse, stimulant use, and marijuana use with a focus on effects on young brains. These events were structured as Native talking circles to discuss issues and arrive at a mutual understanding or agreement [17]. To create a safe environment for discussion, NDN leaders emphasized being respectful, nonjudgmental, and keeping comments confidential. To promote comfort with joining the discussion, NDN leaders started by describing their own experiences with SUD and overdose. Separate meetings with adolescents helped their comfort with discussing sensitive topics and keeping their experiences confidential. The adolescents focused especially on the highly prevalent use of cannabis and addressing traditional practices on reservations promoting its use.

### Measures to Prevent COVID-19 Infection

In the early months of the COVID-19 pandemic, two Wellness Tour community events were conducted remotely using video conferencing supplemented by socially distanced discussions with community members using a speaker. A mutual decision between the NDN and tribal leaders moved these events to in-person to increase the engagement of community members through culturally congruent activities and peer discussions. Safer interactions were facilitated by masking, social distancing, and disclosure of possible COVID-19 symptoms. Some tribes required a negative COVID-19 test for attendance. The first seven sessions also offered live-streaming on Facebook to engage those who wished to minimize their risk of COVID-19 infection. No positive cases were reported to the NDN after each Wellness Tour.

### Data Analysis

A paper questionnaire was administered at eleven Wellness Tours to participants ages 13 years or older; additional questions were included over the course of the Wellness Tour, resulting in varied numbers of responses. A free T-shirt was offered as an incentive for completion.

The questionnaire asked about demographic characteristics (i.e., gender, age, zip code, tribal affiliation) (Table 2) and binary (Y/N) items queried about experiences with substance use or naloxone (Table 3). Added binary (Y/N) questions asked about personal drug use and questions about substance use as follows: “Marijuana (cannabis)”, “Pills”, “Marijuana”, “Alcohol”, “Speed”, “Meth”, “Heroin”, “PCP”, “Molly”, “Opioids” or “I never used drugs”. The generic “pills” option was included to cover any illicit use of drugs in pill form. “Speed” and “Meth” referred to amphetamines and methamphetamines, respectively, while “Molly” referred to Ecstasy.

**Table 2:** Demographic characteristics of respondents to Wellness Tour Survey

Characteristic (N respondents)	
Age, year categories (341)	(%)
0–13	7.6
14–25	21.7
26–35	17.3
36–45	23.2
46–55	15
55+	15
Sex (207)	
Male	34.3
Female	65.7

**Table 3:** Wellness Tour Participant Responses on Past Substance Use

Questions (N respondents)	% Agree
Had access to naloxone before the event (327)	47.0
Ever been offered illicit drugs (52)	86.5
Know someone who overdosed (52)	92.0
Ever used drugs (52)	69.3
Marijuana (cannabis)	92.3
Pills	40.3
Opioids	13.5
Methamphetamines (“Meth”)	52.7
Ecstasy (“Molly”)	19.1
Other (PCP, cocaine, amphetamines, heroin)	16.7

Five-point Likert type questions assessed agreement with statements about the Wellness Tour program (1 = strongly disagree to 5 = strongly agree). Lastly, a 10-point Likert-type question asked about satisfaction with the event, ranging from “not at all satisfied” to “extremely satisfied”. The data were entered into Excel to calculate the frequencies of responses.

## Results

**Fig. 2:**
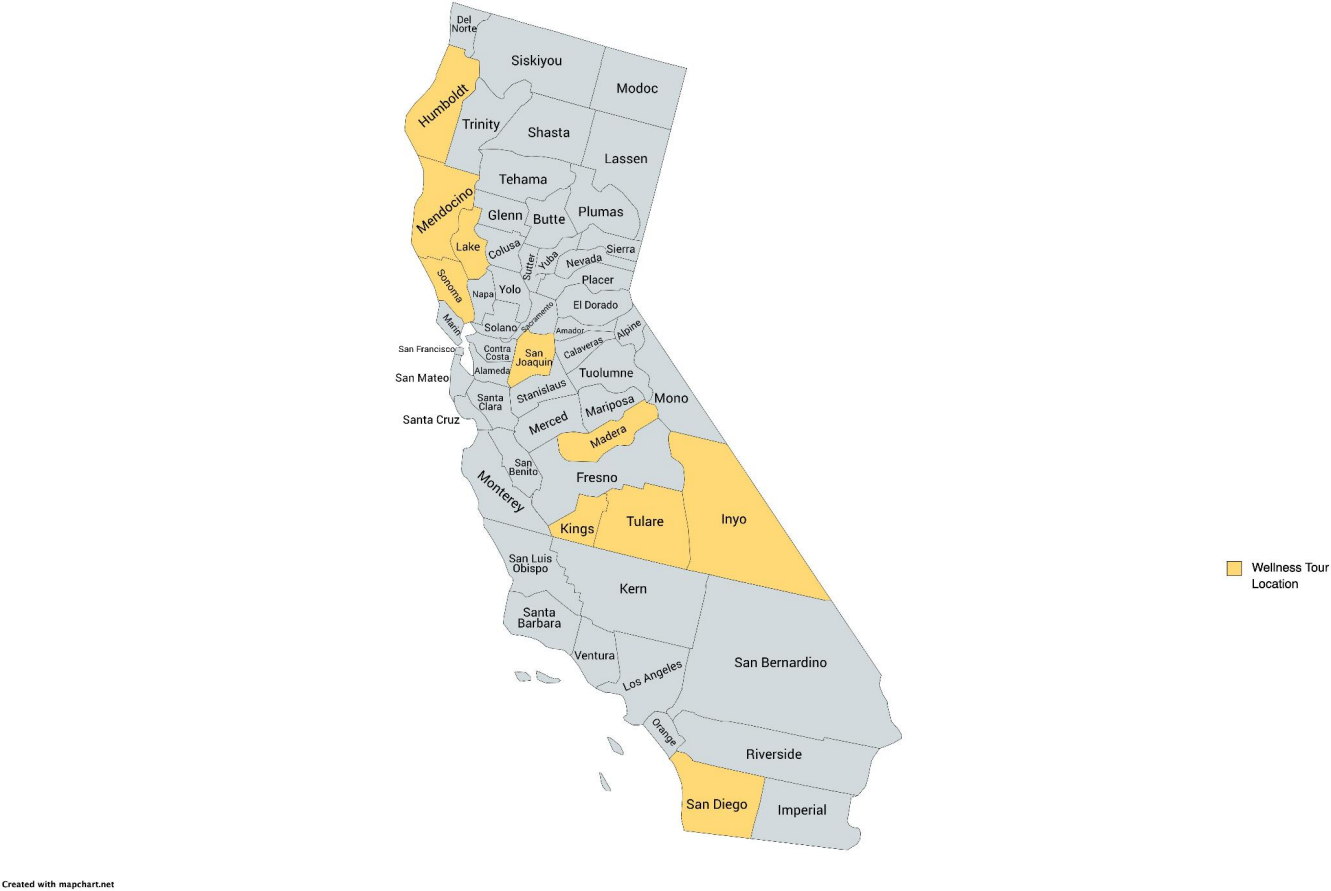
Counties where Wellness Tours were held

From March 2021 through June 2022, 11 tours were held on or near reservations in the following 10 California counties across Northern and Southern California: San Diego, Sonoma, Lake, Madera, Mendocino, San Joaquin, Kings, Tulare, Humboldt, and Inyo (Fig. 2). Wellness Tour attendance ranged from 20 to 168 participants per reservation, with an estimated average of 50 participants.

The anonymous questionnaire was completed by 327 respondents. The majority of participants were aged 34–45 years (23.2%) followed by the 14–25 years age group (21.7%) (Table 2). Of 207 responses regarding sex, two-thirds were women. Members of more than 20 tribes attended the program (not shown).

Slightly less than half of the 327 respondents reported having access to naloxone prior to the Wellness Tour community event (Table 3). Among the 52 participants who answered questions about personal substance use; 86.5% had been offered illicit drugs and 92% knew someone who had overdosed. Approximately two-thirds of the 52 participants admitted to personal illicit drug use which was predominantly marijuana (92.5%) but over half endorsed using methamphetamines while 40% used “pills” (Table 3).

The questionnaire evaluated responses after the Wellness Tour program. On a 10-point satisfaction scale from “Not at all” to “Highly satisfied”, 341 respondents gave a mean rating of 9.7 (SD 2.5) (). Other questions were asked on a five-point scale with a high rating of 4.7 (1 = strongly disagree to 5 = strongly agree) about the program improving the overall quality of life (mentally, emotionally, physically, and spiritually) (Table 4). Ratings for the Wellness Tour were at least 4.6 or higher for being culturally relevant, helping to understand how to use naloxone, and knowledgeable, helpful information from facilitators. A lower 2.9 rating was given by participants for having more NDN events.

**Table 4:** Questions regarding the Native Dads’ Network (NDN) Wellness Tour

Questions (N respondents)	Agreement Mean Responses (SD)
NDN helped to improve my overall quality life (mentally, emotionally, physically, spiritually) (341)	4.7 (0.1)
Noticed an increase in substance use due to the pandemic (243)	4.6 (0.2)
NDN event was culturally relevant (243)	4.8 (0.9)
Understand how to use naloxone and confident can use in an emergency (243)	4.8 (0.9)
The facilitators’ information was helpful, educational and knowledgeable (168)	4.9 (0.6)
Would like to see more NDN events (227)	2.9 (0.8)

* Responses to Likert-scale (1 = strongly disagree to 5 = strongly agree)

Information about the risk of contracting COVID-19 infection was available only for the NDN leaders and their families, none of whom developed COVID after meetings. Informal follow-up with communities did not identify positive cases occurring shortly after the meetings.

## Discussion

The Wellness Tour was developed and implemented by AIAN community leaders during the COVID-19 pandemic in response to the dire impact of SUD in their tribes and personal lives. The AIAN population had the greatest increase in drug overdose deaths from 2018 to 2021, the first year of the pandemic, according to National Vital Statistics [18]. The Wellness Tour reached 11 tribal communities across California at a time when they were even more isolated due to the pandemic. It was specifically designed to be culturally appropriate for AIAN communities by delivering SUD prevention education in conduction with talking circles, skits, and the creation of “sacred space” through prayer, song, and dance. Cultural spirituality and prayer are key features of SUD prevention for AIAN communities and have been reported to improve mental health status in AIAN persons [19, 20].

Uniquely, nine of the 11 Wellness Tour meetings were held in person, despite the COVID-19 pandemic, to increase the engagement of AIAN communities. NDN leaders often traveled great distances to deliver the program at or near diverse tribal reservations, many of which were in remote rural locations. In-person meetings featured ceremonial activities to promote a culturally supportive, trusting environment with sharing of sensitive experiences that might have been more challenging using a video platform. These activities called on cultural values and traditions that have been strongly endorsed in focus groups with AIAN adolescents [21] and adults [22]. The Wellness Tours’ structure reflected studies in AIAN populations supporting the need for community and family engagement; cultural practices based on AIAN experience; respect for clients; and a homelike atmosphere [23]. A focus on respect and cultural competence has been reported to be protective against SUD in AIAN communities by reinforcing spiritual identity, positive health behaviors, and community cohesiveness as well as promoting wellness [23]. The questionnaire completed after the program by over 300 attendees indicated respondents strongly agreed with the statement that the NDN [leaders] helped to improve the overall quality of life, supporting a highly favorable response to this program designed specifically for AIAN communities.

Many SUD prevention programs include adolescents and young adults because they are a high risk group [24]. NDN leaders elected to engage all age groups but offered unique opportunities for younger attendees to participate such as acting in a skit about using naloxone and holding separate talking circles from adults to facilitate more frank and open discussions about drug use. Such culturally aligned activities have been reported to reduce the risk of substance use in AIAN youth as well as suicide [25]. Another recent study of 288 tribal adolescents and young adults reported that the adjusted odds of polysubstance abuse was reduced by over 30% for those who had a strong tribal identity [19].

The NDN leaders were all AIAN and personally knowledgeable about SUD as counselors or being personally affected by SUD in their families. Respondents gave the program high ratings on cultural relevance and on NDN leaders as knowledgeable educators. These results lend support to the success of NDN leaders in engaging diverse tribes across the State.

The Wellness Tour incorporated Western medicine-based education, medication, and testing to address SUD, especially about opioids, capitalizing on substantial research and national initiatives reporting reduced risk for drug overdose and death [26–28]. The program bolstered the SUD educational program with training and distribution of free naloxone and fentanyl test strips that have not been widely available to AIAN communities. The Department of Environmental Health distributed naloxone only to first responders in Southwestern US AIAN in 2021 [29]. A new initiative to offer naloxone over the counter may help address this deficit but still requires pharmacies to supply the medication and incurs a cost [30].

We acknowledge the limitations of this community-based study. First, respondents to the post-meeting questionnaire represent a convenience sample and questions were added to the questionnaire over the course of the Tour. Second, we lack information about changes in substance use behaviors after the program. Third, we have only informal information about COVID-19 transmission among participants. In support of belief in its safety, across the state AIAN tribal community leaders continued to invite the NDN to hold the program during the pandemic. Fourth, the sustainability of the Wellness Tour is unclear given substantial travel and time required of the NDN leaders as well as reliance on grant funding along with food and lodging donated by several reservations. In the future, a cost-effectiveness analysis should be conducted to support investment in Wellness Tours. However, the questionnaire responses suggested that the Tour did not have to reach the same tribes again but many AIAN communities have not received this program. Lastly, the Wellness Tour educational program focused especially on opioid abuse but other forms of substance abuse were addressed informally during the talking circles. Future community-based programs should include broader education about drugs and alcohol given polysubstance abuse described by respondents on the post-meeting survey.

Mistrust of Western-medicine by AIAN communities [31] reinforces the role of AIAN-led organizations to deliver community-based, culturally congruent SUD prevention education and support. Notably, several hundred participants in the Wellness Tour program reported that it improved their general well-being. The diverse AIAN tribal communities reached by the Wellness Tour and its broad engagement of youth and adults offers a potentially valuable model to deliver culturally appropriate SUD prevention to reduce the disastrous impact of illicit drug use on communities.
